# The effect of essential oils and their combinations on bacteria from the surface of fresh vegetables

**DOI:** 10.1002/fsn3.1864

**Published:** 2020-09-03

**Authors:** Éva György, Éva Laslo, Ildikó Hajnalka Kuzman, Csaba Dezső András

**Affiliations:** ^1^ Department of Food Science Faculty of Economics Socio‐Human Sciences and Engineering Sapientia Hungarian University of Transylvania Miercurea Ciuc Romania; ^2^ Department of Bioengineering Faculty of Economics Socio‐Human Sciences and Engineering Sapientia Hungarian University of Transylvania Miercurea Ciuc Romania

**Keywords:** antimicrobial effects, essential oils, foodborne pathogenic bacteria, fresh vegetables, spoilage bacteria

## Abstract

During the study, we determined the antimicrobial activity of different selected essential oils (thyme, lemongrass, juniper, oregano, sage, fennel, rosemary, mint, rosehips, dill) on some pathogenic and spoilage bacteria isolated from the surface of various fresh vegetables. At the same time, in the case of some volatile oil combinations we followed the phenomena of synergism and antagonism. The identification of the isolated bacterial strains was made using 16S rDNA gene sequence analysis. The most resistant isolates appeared to be *Curtobacterium herbarum*, *Achromobacter xylosoxidans,* and *Enterobacter ludwigii*, while *Pseudomonas hibiscicola* was the most sensitive. Of the chosen plant essential oils, the most pronounced antimicrobial effect was detected in the case of oregano. The essential oils of thyme and mint also showed elevated antimicrobial activity. A synergistic effect was observed in case of five combinations of essential oil. Based on the results, we find that some individual essential oils and mixture compositions (due to synergic effect) could be good candidates for the preservation of fresh vegetables. These preliminary findings suggest that essential oils from locally grown spices could contribute to decreasing the health risk and also to the suppression of emergence of antibiotic resistance.

## Graphical abstract

Is very useful to know the pathogenic strains occurrence in different geographic regions, to prevent disease occurrence in population and among the tourists, how are more susceptible to foodborne pathogens. The main goal was the detection of active antimicrobial essential oil compositions that will be used as ingredients in active packaging materials for fresh vegetables and fruits, for decreasing the health risk and increasing the product shelf‐life.

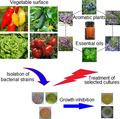

## 1 INTRODUCTION

Different parts of vegetables are edible, such as roots, tubers, leaves, flowers, and bulbs. Generally, these plant components are rich in carbohydrates, with pH values ranging from 5.0 to 7.0. In favorable conditions, different microorganisms such as bacteria, yeasts, and molds can colonize them. The main source of microbes in vegetables includes air, soil, water, domestic or wild animals, insects, or different equipments. Microorganisms can differ with the types of vegetables (Ray & Bhunia, 2014). The microbiota associated with most vegetables is dominated by the Gram‐negative bacteria. In the case of many vegetables, *Pseudomonas* spp. dominate the microflora and normally represent 50%–80% of the microbial population (Ramos, Miller, Brandão, Teixeira, & Silva, 2013). In the development of the food spoilage microbial community on the vegetable surface (beside external environmental factors), the prevailing internal properties of the vegetables (chemical composition, water activity, pH‐value, redox potential) play an important role. The physiological state of the plant is an important factor in resistance to the microbial spoilage. The main cause of spoilage of fresh vegetables is bacteria, where their proliferation rate exceeds the growth rate of the yeasts and molds, particularly in a cooled environment. Among the Gram‐negative bacterial strains *Acinetobacter*, *Chromobacterium*, *Alcaligenes*, *Enterobacter*, *Flavobacterium*, *Klebsiella*, *Pseudomonas*, *Serratia*, and *Xanthomonas* species can often be isolated. The most common Gram‐positive bacteria that occur on vegetables are *Micrococcus*, *Lactobacillus*, *Corynebacterium*, and *Bacillus* species (Deák, 2006). At refrigeration temperature storage, the dominant bacterial populations on the surface of minimally processed vegetables belong to different families such as *Pseudomonadaceae*, *Enterobacteriacae*, and also different species of lactic acid bacteria. The most representative species are *P. fluorescens, Rahnella aquatilis, Erwinia herbicola*, and *Leuconostoc mesenteroides* (Prakash, Baskaran, Paramasivam, & Vadivel, 2018). Enteric pathogens from vegetables are a public health concern. In general, transmission of these pathogens is possible from manure contaminated soil, feces of animals and birds, irrigation water (Adams & Moss, 2008). Major pathogen bacteria from vegetables associated with outbreaks are as follows: *Clostridium botulinum*, *E. coli* O157:H7, *Listeria monocytogenes*, *Salmonella* spp. *Shigella* spp., *Staphylococcus* spp., *Vibrio cholerae*, and *Yersinia enterocolitica* (Cayalvizhi & Balachandhar, 2019; Ramos et al., 2013).

Minimally processed vegetables are one of the largest rising sectors in food industry. Ongoing advancement in natural food preservatives has led to the use of essential oils or their components in food packaging, and they represent an alternative to the synthetic additives (Patrignani, Siroli, Serrazanetti, Gardini, & Lanciotti, 2015). Essential oils have exhibited antagonistic properties against different pathogenic or spoilage microorganisms. In vitro studies have shown antibacterial activity of essential oils against *Bacillus cereus, Escherichia coli, Listeria monocytogenes, Salmonella typhimurium,* and *Staphylococcus aureus* with concentration ranging from 0.2 to 10 μl/ml (Scollard, Francis, & O'Beirne, 2013). Some essential oils such as *Citrus* spp., cinnamon, oregano, and thyme have been utilized as natural antimicrobials in different food preparations, while uncommon, plant‐derived essential oils have received minimal attention. For example, *Thymbra capitata* essential oil, containing mainly carvacrol (93.31%) as oxygenated monoterpenes, exhibited strong antimicrobial activity. It was assayed as a potential sanitizing solution in food industry for food contact surfaces (Falcó, Verdeguer, Aznar, Sánchez, & Randazzo, 2019). Essential oils of *Azadirachta indica* and *Litsea cubeba* were recognized as promising and successful new antimicrobials with significant antimicrobial activity against the *Staphylococcus aureus* and *Escherichia coli*, concerning possible applicability in food (Thielmann, Muranyi, & Kazman, 2019). Subinhibitory concentrations of *Origanum vulgare* and *Rosmarinus officinale* combined essential oils effectively inhibited the growth and survival of different pathogenic (*Listeria monocytogenes*, *Yersinia enterocolitica*, *Aeromonas hydrophila*) and spoilage (*Pseudomonas fluorescens*) bacteria related to minimally processed vegetables. Sensory evaluation showed that consumers find it acceptable for the different essential oils to be used together at subinhibitory concentrations as vegetable sanitizer. These forms of essential oils may contribute to the extension of the storage and shelf‐life of vegetables (Azeredo et al., 2011). The effectiveness of essential oil combination is associated with the increased membrane damages showing a stronger antimicrobial effect (Yuan, Teo, & Yuk, 2018). A recent study proposes the use of essential oils as natural antibacterial inhibitors in nanoemulsion formulations against foodborne pathogens isolated from fresh fruits and vegetables (Amrutha, Sundar, & Shetty, 2017). The microbial quality and safety of minimally processed vegetables have been remarkably improved with the application of essential oil‐based nanoemulsions. These formulations were used as washing disinfectant or incorporated into edible coatings on packaging (Dvir et al., 2019) or product surface (Prakash et al., 2018). A new application is small concentrations of cinnamon essential oil, which are capable of reducing the attachment of *Salmonella* strains to the lettuce surface during refrigerated storage, with a remarkably positive effect on food safety (Rossi et al., 2019).

For the prevention of foodborne disease occurrence, it could be useful to know the specific pattern of pathogenic strains in different geographic regions. Susceptibility of these bacteria to essential oils could be a good candidate for combating them using antimicrobial packaging, without risk of the emergence of antibiotic resistance. In this research, we determined the antimicrobial effect of different essential oils and certain combinations of essential oils, with the agar diffusion method on pathogenic as well as spoilage bacteria isolated on selective culture media and identified, from the surface of various fresh vegetables.

## 2 MATERIALS AND METHODS

The first step of our research was to examine the microbial contamination of the surface of various fresh vegetables (cucumber, lettuce, tomatoes, peppers, hot peppers, cabbage, radishes, broccoli, and onions) with cultivation methods on different selective media. Pseudomonas isolation agar base was used to detect the presence of *Pseudomonas* species. TBX Chromo Agar was used for the detection and enumeration of *Escherichia coli*. XLD Agar and BPLS agar were used for the detection of *Salmonella* species. Campylobacter‐Blood Free Selective Agar was used for the detection of *Campylobacter* species. TCBS Agar was applied for the detection of *Vibrio* species, for the detection of *Staphylococcus* sp. Mannitol Salt Agar was used. Cereus Selective Agar was applied for the detection of *Bacillus cereus*, and Listeria mono Differential Agar was used for the detection of *Listeria* species.

The vegetables were purchased from the local market. The bacterial strains occurring at highest amount on selective media were identified by molecular biological method. Subsequently, the antibacterial activity of a variety of individual herbal essential oils and certain combinations of essential oils were studied by agar diffusion method in the case of nine isolated and identified bacterial species: *Pseudomonas hibiscicola, Brevibacillus agri, Enterobacter ludwigii, Curtobacterium herbarum, Acinetobacter beijerinckii, Acinetobacter calcoaceticus, Achromobacter xylosoxidans, Staphylococcus succinus, and Staphylococcus sciuri*. Ten commercially available essential oils were used in this study: thyme, lemongrass, juniper, oregano, sage, fennel, rosemary, mint, rosehips, and dill. These oils were selected based on their herbal use in traditional culinary practices. These oils were produced by different companies (*Solaris Plant*, *Adams Vision, Herbavit, Aromax*, *Fares Bio Vital*, and *Hofigal Export‐Import*) and extracted from different parts of the herbs.

The identification of the isolated bacterial strains was obtained using 16S rDNA sequence analysis. AccuPrep^®^ Genomic DNA Extraction Kit from Bioneer was used for Genomic DNA isolation. Isolations were performed according to the manufacturer's protocol. For the amplification of one part of the bacterial 16S rDNA gene, universal oligonucleotides were used 27f and 1492r (5’ AGAGTTTGATCMTGGCTCAG 3’, 5’ TACGGYTACCTTGTTACGACTT 3’). The PCR program for amplification was as follows: an initial denaturation at 94°C for 5 min, which was followed by 30 cycles of denaturation at 94°C for 30 s, annealing at 55°C for 30 s, extension at 72°C for 1 min, and a final extension at 72°C for 7 min. Sequencing was performed by Biomi KFT (Hungary). The sequences were edited and aligned using Chromas (Technelysium Pty. Ltd., South Brisbane, Australia); Molecular Evolutionary Genetics Analysis 4 system was used for the phylogenetic analyses. The isolates were identified through comparison of the sequences using the EzTaxon server on the basis of 16SrDNA sequence data (Laslo, György, Ábrahám, & Mara, 2017).

In the agar diffusion method, 20 ml nutrient agar medium was poured in a sterilized Petri dish. After solidification, the medium was inoculated on the surface with a 0.1 ml suspension of bacteria (10^8^ CFU/ml) taken in study. In the center of all of the inoculated media, an 8 mm diameter hole was cut with the help of a sterile test‐tube. In this hole 0.05 ml of essential oil was dropped. The incubation was carried out at the temperature of 30ºC, 48 hr. In the case of the essential oil combinations, in the holes made in the inoculated media, 0.025 ml essential oils were added. After incubation, the results were read and expressed in accordance with the size of the inhibition zone (György, Laslo, & András, 2015).

The statistical analysis was performed with Statistica 8.0 (StatSoft, Inc., Oklahoma, USA).

## 3 RESULTS AND DISCUSSION

Among the studied vegetables, in the case of the lettuce, cucumber, radishes, and onions, the contamination was higher compared to the other vegetables. The results show that the microbiota occurring on the surface of fresh vegetables is highly variable and the molecular biological studies have identified a variety of pathogenic, saprophytic, and food spoilage bacteria (Table 1). It is important to highlight that the majority of the isolated and identificated bacteria during our research are not commonly found on the vegetable surfaces. It has been shown that different factors contribute to the diversity and composition of bacterial communities associated with the surfaces of fresh vegetables (Leff & Fierer, 2013).

**TABLE 1:** Results of the identification of the bacterial strains isolated from the surface of different fresh vegetables

Most closely related organism	Source of isolation	% Gene identity
*Achromobacter xylosoxidans*	Radish	99.99
*Acinetobacter calcoaceticus*	Radish	100
*Acinetobacter beijerinckii*	Lettuce	99.71
*Bacillus thuringiensis berliner*	Onion	98.32
*Bacillus subtilis* subsp. *inaquosorum*	Cabbage	98.24
*Brevibacterium frigoritolerans*	Broccoli	67
*Brevibacillus agri*	Radish	98.77
*Cellulomonas pakistan* *e* *nsis*	Onion	90.07
*Curtobacterium herbarum*	Cabbage	98.95
*Curtobacterium herbarum*	Onion	87.42
*Enterococcus casseliflavus*	Broccoli	99,69
*Enterococcus casseliflavus*	Cucumber	98.35
*Enterobacter ludwigii*	Radish	99.8
*Enterobacter ludwigii*	Radish	99.48
*Pseudomonas geniculate*	Cucumber	98.55
*Pseudomonas hibiscicola*	Cucumber	99.9
*Pseudomonas hibiscicola*	Tomato	98.98
*Pseudomonas hibiscicola*	Cucumber	99.19
*Pseudomonas hibiscicola*	Cucumber	99.17
*Pseudomonas hibiscicola*	Cucumber	99.15
*Pseudomonas monteilii*.	Lettuce	99.61
*Pseudomonas putida*	Cucumber	98.49
*Pseudomonas oryzihabitans*	Cucumber	99.45
*Pseudomonas oryzihabitans*	Cucumber	98.94
*Staphylococcus succinus* subsp. *succinus*	Red pepper	99.85
*Staphylococcus sciuri* subsp. *sciuri*	Tomato	99.9
*Staphylococcus sciuri* subsp*. sciuri*	Tomato	99.7
*Staphylococcus sciuri* subsp. *sciuri*	Tomato	100
*Staphylococcus sciuri* subsp. *sciuri*	Red pepper	98.87
*Stenotrophomonas maltophilia*	Lettuce	98.78

The identified bacterial strains were isolated from the used selective media as follows: a *Pseudomonas hibiscicola* from Mannitol Salt Agar; another *Pseudomonas hibiscicola, P. putida*, and *P. monteilii* from Pseudomonas isolation agar base; *Brevibacillus agri* and *P. geniculata* from BPLS agar (for the isolation of *Salmonella*); *Enterobacter ludwigii* and *Achromobacter xylosoxidans* from XLD Agar; *Curtobacterium herbarum, Enterococcus casseliflavus*, and *Cellulomonas pakistanensis* from Listeria mono Differential Agar; *Acinetobacter beijerinckii* and *Acinetobacter calcoaceticus* from Campylobacter‐Blood Free Selective Agar; *Staphylococcus succinus* and *Staphylococcus sciuri* from Mannitol Salt Agar, *Bacillus thuringiensis berliner, Bacillus subtilis* subsp. *inaquosorum,* and *Brevibacterium frigoritolerans* from Cereus Selective Agar; and *Pseudomonas oryzihabitans* from TBX Chromo Agar.

According to the result of the 16S rDNA sequence analysis, the bacterial isolates belong to 11 genera, showing 98%–100% similarity, with the exception of three bacterial isolates. In these cases, the gene identity is below 98%.

The most commonly occurring species was *Pseudomonas hibiscicola*. *Pseudomonas* genus includes large numbers of species. The majority of spoilage bacteria can transform due to the metabolization wide variety of food components as carbohydrates, proteins, and lipids. The *Pseudomonas hibiscicola* bacterium was firstly isolated by Monis in 1963, but further research shows that this strain can be found in the microbial nomenclature also known as *Xanthomonas maltophilia* and *Stenotrophomonas maltophilia*. On the basis of the phylogenetic tree of the γ and the γ‐β subclasses of the Proteobacteria derived from the similarities of the 16S rDNA sequence, *Pseudomonas hibiscicola* belong to the *Xanthomonas* group, next to *Stenotrophomonas maltophilia* (Anzai, Kim, Park, Wakabayashi, & Oyaizu, 2000). Bacterial strain designated SD8 was isolated from sea muds of the Qinhuangdao sea area in China. This marine bacterial strain was able to produce alkaline protease approximatively at a low yield. The bacterial strain was identified as *Pseudomonas hibiscicola* based on 16S rDNA sequence analysis and morphological, physiological, and biochemical characterization (Cui, Yang, Wang, & Xian, 2015). *Pseudomonas hibiscicola* was isolated from ticks (Murrell et al., 2003). This bacterium is naturally occurring on the surface of roots, in the soil, and is an opportunistic plant pathogen. Other isolated *Pseudomonas* species were as follows: *Pseudomonas oryzihabitans, P. geniculata, P. putida*, and *P. monteilii*. Other isolated bacteria with highest occurrence on the surface of fresh vegetables were as follows: *Acinetobacter beijerinckii, A. calcoaceticus, Achromobacter xylosoxidans, Bacillus thuringiensis berliner, Bacillus subtilis* subsp. *inaquosorum, Staphylococcus sciuri* subsp*. sciuri, Staphylococcus succinus* subsp. *succinus, Enterococcus casseliflavus, Enterobacter ludwigii, Brevibacillus agri, Brevibacterium frigoritolerans, Curtobacterium herbarum, Cellulomonas pakistanensis,* and *Stenotrophomonas maltophilia*.

Based on the results, the antimicrobial activity of essential oils shows differences in the function of bacterial strains. The higher efficiency was observed for oregano essential oil, when total inactivation was observed for some strains (*Pseudomonas hibiscicola, Brevibacillus agri*), and for the other studied strains, the formation of large inhibition zone was observed. The highest number of *Brevibacillus* strains have been isolated from different natural environments. The origin of these strains is soils, where they occur as saprophytes. Some strains have also been isolated from human clinical samples and from human diseases. *Brevibacillus agri* is an aerobic, motile, oxidase negative, catalase positive, Gram‐positive bacteria with rod shaped cell morphology, isolated from soil, water, sterilized milk, clinical specimens, pharmaceutical manufacturing plants, and a public water supply where it was implicated in an outbreak of waterborne illness (Vos et al., 2009).

The essential oils of thyme and mint also show elevated antimicrobial activity (Table 2). The mint essential oil determined total inhibition to *Pseudomonas hibiscicola*, *Enterobacter ludwigii,* and *Achromobacter xylosoxidans*. *Enterobacter ludwigii* strains are motile rods, Gram‐negative bacteria. These strains are characterized by the possession of catalase, oxidase, and the lack of DNAase. They are fermentative bacteria and nonpigmented. They exhibit the same general characteristic as the family *Enterobacteriaceae*, the genus *Enterobacter*, and the *E. cloacae* complex. The capacity to grow on myo‐inositol and 3–0‐methyl‐D‐glucopyranose can differentiate the *E*. *ludwigii* from the other species of the genus (Hoffmann et al., 2005). *Enterobacter* species, such *Enterobacter ludwigii*, can cause different infections connected to abdominal, urinary tract, meningeal, and surgical sites (Khajuria, Praharaj, Grover, & Kumar, 2013). *Achromobacter xylosoxidans* belong to the Alcaligenaceae family. This strain is an aerobic characterized by motility, nonfermenting Gram‐negative rod. This bacterium is oxidase—and catalase positive, and is ubiquitous in aqueous environments. *A. xylosoxidans* is generally considered as an opportunistic microorganism with low virulence. It is most often isolated from humans. It was detected in adults and neonates suffering from simultaneous chronic diseases comorbidities and from indwelling medical devices (Claassen, Reese, Mysliwiec, & Mahlen, 2011). Primarily, it has also been detected in different infectious etiologies as in immunocompromised individuals. A case was also reported where *A*. *xylosoxidans* caused osteomyelitis in a patient with diabetes mellitus. Generally, this bacterium is characterized by multidrug resistance (Shinha & Oguagha, 2015). In patients with skin and soft tissue infections or with vascular diseases, *A. xylosoxidans* should be treated as potential pathogen. In humans, after surgery or trauma, pathogenic bacteria should also be considered (Tena, Martínez, Losa, & Solís, 2014).

**TABLE 2:** The effect of the essential oils on growth of the studied bacteria I. (Inhibition zone in mm, average ± *S.D*., *n* = 10)

Studied bacteria	Thyme	Oregano	Mint	Lemongrass	Sage
*Pseudomonas hibiscicola*	Total inhibition	Total inhibition	Total inhibition	10.2 ± 0.20	2.8 ± 0.20
*Brevibacillus agri*	Total inhibition	Total inhibition	3.4 ± 0.24	10.6 ± 0.24	3.8 ± 0.37
*Enterobacter ludwigii*	9.0 ± 0.0	18.2 ± 1.07	Total inhibition	2.0 ± 0,0	No inhibition
*Curtobacterium herbarum*	13.2 ± 1.39	19.2 ± 0.80	1.0 ± 0.0	1.0 ± 0.0	No inhibition
*Acinetobacter beijerinckii*	22.8 ± 0.20	17.6 ± 0.81	4.8 ± 0.20	7.0 ± 0.0	1.0 ± 0.0
*Acinetobacter calcoaceticus*	41.2 ± 1.11	27.4 ± 1.25	3.6 ± 0.24	3.2 ± 0.20	3.0 ± 0.0
*Achromobacter xylosoxidans*	4.8 ± 0.37	11.4 ± 0.51	Total inhibition	2.0 ± 0.0	No inhibition
*Staphylococcus succinus*	23.6 ± 1.81	31.2 ± 4.37	1.4 ± 0.24	5.6 ± 0.24	1.0 ± 0.0
*Staphylococcus sciuri*	11.2 ± 0.20	20.0 ± 2.12	2.6 ± 0.24	8.0 ± 0.0	2.4 ± 0.24

The sensitivity of bacterial strains isolated from the surface of fresh‐cut vegetables relative to other selected essential oils was low or even shows lack of effects. The weakest activity on bacteria was shown by rosehips and sage essential oils (Tables 2 and 3).

**TABLE 3:** The effect of the essential oils on growth of the studied bacteria II. (Inhibition zone in mm, average ± *S.D*., *n* = 10)

Studied bacteria	Rosemary	Fennel	Juniper	Dill	Rose hips
*Pseudomonas hibiscicola*	6.5 ± 0.39	4.0 ± 0.45	5.4 ± 0.24	5.6 ± 0.40	4.1 ± 0.40
*Brevibacillus agri*	5.7 ± 0.30	1.4 ± 0.24	5.0 ± 0.45	12.2 ± 1.74	No inhibition
*Enterobacter ludwigii*	9.4 ± 0.40	1.0 ± 0.0	6.6 ± 1.05	No inhibition	No inhibition
*Curtobacterium herbarum*	13.0 ± 0.0	1.0 ± 0.0	No inhibition	2.0 ± 0.0	No inhibition
*Acinetobacter beijerinckii*	8.8 ± 1.96	7.8 ± 0.37	6.2 ± 0.37	Total inhibition	No inhibition
*Acinetobacter calcoaceticus*	7.0 ± 0.0	3.6 ± 0.24	9.2 ± 0.86	18.0 ± 0.45	No inhibition
*Achromobacter xylosoxidans*	5.6 ± 0.24	No inhibition	No inhibition	No inhibition	1.0 ± 0.0
*Staphylococcus succinus*	14.8 ± 0.37	1.0 ± 0.0	3.6 ± 0.4	1.0 ± 0.0	No inhibition
*Staphylococcus sciuri*	11.6 ± 0.24	2.4 ± 0.24	5.6 ± 0.40	2.0 ± 0.0	No inhibition

In case of the dill essential oil, total inactivation was observed for *Acinetobacter beijerinckii. Acinetobacters* are present naturally in soil and water and occur in sewage. They have been detected in different foods such as raw, washed, and frozen vegetables, and they have also occurred in fresh, frozen, and stored fish products, as well as in spoiled animal‐origin foods such as meat, milk, and cheese (Brenner, Krieg, & Staley, 2005). Different strains of *Acinetobacter beijerinckii* were isolated from human clinical samples (Nemec et al., 2009), from ready to eat fruit and lettuce (Carvalheira, Silva, & Teixeira, 2017).

The most sensitive bacterium against essential oils was *Pseudomonas hibiscicola*, and the most resistant was *Curtobacterium herbarum*. Strains belonging to *Curtobacterium* have been isolated from different plants and rice, and *C. herbarum* was isolated from grass. *Curtobacterium* strains are rarely isolated from clinical specimens. It is recommended for clinical microbiologists to be aware of the possible occurrence of these bacteria in human samples. Because of the everyday exposure of people to *Curtobacterium* their pathogenicity is considered rather low (Funke, Aravena‐Roman, & Frodl, 2005).

Based on the results, the most resistant strain against the essential oil combinations was the *Curtobacterium herbarum*. The essential oils exert only a small antimicrobial effect on them (the most effective was the oregano essential oil), and in 7 cases, a lack of inhibition was observed (Tables 4, 5, 6 and 7). Similar results were obtained for the strain *Achromobacter xylosoxidans*, 7 essential oil combinations did not inhibit them, and generally a slight antibacterial effect was observed. The *Enterobacter ludwigii* strain shows resistance to essential oils, since in most cases, minimal inhibition was detectable (likewise, the oregano was the most effective). However, in this last two cases, the combination of thyme and dill essential oils showed synergism. The most susceptible to single essential oil inhibition was the bacteria *Pseudomonas hibiscicola*, alongside the essential oil combinations. In case of five combinations of essential oil, synergistic effect was observed (i.e., a larger inhibition zone was observed for combinations in comparison with the effect of the individual essential oils). The synergistic combinations were as follows: lemongrass‐rosemary, sage‐rosemary, dill‐rosemary, juniper‐cumin, and cumin‐dill. For the bacterial strain *Acinetobacter calcoaceticus* juniper‐cumin essential oil combination show an enhanced antibacterial effect. Species belonging to the *Acinetobacter calcoaceticus*–*Acinetobacter baumannii* complex group are believed to be nosocomial pathogens. This group of bacteria is nonfermenting, aerobic, and Gram‐negative coccobacilli (Lai et al., 2012).

**TABLE 4:** The effect of the combination of essential oils on the studied bacteria I. (Inhibition zone in mm, average ± *S.D*., *n* = 10)

Studied bacteria	Lemongrass + Juniper	Lemongrass + Fennel	Lemongrass + Sage	Lemongrass + Rosemary	Lemongrass + Rose hips
*Pseudomonas hibiscicola*	11.2 ± 0.58	8.8 ± 0.25	5.4 ± 0.24	29.2 ± 5.45	5.2 ± 0.37
*Brevibacillus agri*	4.0 ± 0.45	5.8 ± 0.66	1.4 ± 0.24	4.6 ± 0.24	4.0 ± 0.32
*Enterobacter ludwigii*	3.4 ± 0.87	1.6 ± 0.24	3.2 ± 0.0	5.8 ± 0.58	1.0 ± 0.0
*Curtobacterium herbarum*	No inhibition	1.0 ± 0.0	No inhibition	6.8 ± 0.2	1.0 ± 0.0
*Acinetobacter beijerinckii*	6.6 ± 0.24	9.6 ± 0.51	9.6 ± 0.24	11.2 ± 0.2	8.8 ± 0.37
*Acinetobacter calcoaceticus*	10.2 ± 0.58	3.8 ± 0.2	No inhibition	12.8 ± 0.37	8.0 ± 0.55
*Achromobacter xylosoxidans*	4.8 ± 0.20	1.0 ± 0.0	No inhibition	10.0 ± 0.32	1.0 ± 0.0
*Staphylococcus succinus*	8.0 ± 0.45	4.8 ± 0.37	3.6 ± 0.4	10.0 ± 0.0	6.4 ± 0.4
*Staphylococcus sciuri*	5.8 ± 0.2	6.4 ± 0.24	5.0 ± 0.0	8.0 ± 0.32	5.4 ± 0.24

**TABLE 5:** The effect of the combination of essential oils on the studied bacteria II. (Inhibition zone in mm, average ± *S.D*., *n* = 10)

Studied bacteria	Juniper + Fennel	Juniper + Sage	Juniper + Rosemary	Dill + Fennel	Dill + Sage
*Pseudomonas hibiscicola*	19.8 ± 0.37	3.6 ± 0.40	11.4 ± 0.51	14.2 ± 1.02	1.4 ± 0.24
*Brevibacillus agri*	6.6 ± 0.51	10.8 ± 1.07	2.8 ± 0.20	10.8 ± 1.39	9.6 ± 0.24
*Enterobacter ludwigii*	No inhibition	No inhibition	2.8 ± 0.37	2.4 ± 0.24	2.4 ± 0.24
*Curtobacterium herbarum*	2.8 ± 0.37	2.6 ± 0.24	9.6 ± 0.24	2.6 ± 0.4	1.8 ± 0.2
*Acinetobacter beijerinckii*	10.2 ± 0.49	No inhibition	13.0 ± 0.32	16.2 ± 0.97	26.0 ± 1.55
*Acinetobacter calcoaceticus*	Total inhibition	No inhibition	14.6 ± 0.24	5.6 ± 0.6	15.0 ± 1.41
*Achromobacter xylosoxidans*	1.4 ± 0.24	No inhibition	No inhibition	3.4 ± 0.51	No inhibition
*Staphylococcus succinus*	2.0 ± 0.0	1.0 ± 0.0	5.0 ± 0.0	1.8 ± 0.2	2.8 ± 0.37
*Staphylococcus sciuri*	2.0 ± 0.0	6.8 ± 0.2	9.4 ± 0.24	1.0 ± 0.0	3.2 ± 0.37

**TABLE 6:** The effect of the combination of essential oils on the studied bacteria III. (Inhibition zone in mm, average ± *S.D*., *n* = 10)

Studied bacteria	Dill + Rosemary	Sage + Rosemary	Sage + Fennel	Thyme + Mint	Thyme + Dill
*Pseudomonas hibiscicola*	29.2 ± 1.74	22.4 ± 0.93	10.0 ± 0.71	Total inhibition	29.2 ± 1.74
*Brevibacillus agri*	13.0 ± 0.89	4.8 ± 0.58	2.9 ± 0.10	4.0 ± 0.45	21.6 ± 1.29
*Enterobacter ludwigii*	3.8 ± 0.20	3.6 ± 0.68	No inhibition	3.4 ± 0.24	17.2 ± 1.66
*Curtobacterium herbarum*	9.2 ± 0.2	7.4 ± 0.24	3.2 ± 0.49	4.6 ± 0.24	5.0 ± 0.0
*Acinetobacter beijerinckii*	11.0 ± 0.32	10.0 ± 0.32	2.6 ± 0.24	10.6 ± 0.51	14.4 ± 0.51
*Acinetobacter calcoaceticus*	10.6 ± 0.51	10.8 ± 0.37	1.6 ± 0.24	14.4 ± 0.75	23.0 ± 0.71
*Achromobacter xylosoxidans*	9.2 ± 0.2	9.0 ± 0.32	No inhibition	5.6 ± 0.93	10.0 ± 1.14
*Staphylococcus succinus*	9.4 ± 0.24	8.8 ± 0.2	1.0 ± 0.0	10.0 ± 0.0	7.0 ± 0.95
*Staphylococcus sciuri*	8.2 ± 0.2	9.4 ± 0.24	5.6 ± 0.51	3.0 ± 0.0	2.8 ± 0.2

**TABLE 7:** The effect of the combination of essential oils on the studied bacteria IV. (Inhibition zone in mm, average ± *S.D*., *n* = 10)

Studied bacteria	Thyme + Juniper	Mint + Juniper	Mint + Sage	Oregano + Sage	Rosehips + sage
*Pseudomonas hibiscicola*	26.4 ± 2.46	6.4 ± 1.03	2.4 ± 0.24	10.4 ± 0.75	1.0 ± 0.0
*Brevibacillus agri*	28.8 ± 3.31	4.2 ± 0.73	4.0 ± 0.55	11.0 ± 0.32	No inhibition
*Enterobacter ludwigii*	6.8 ± 0.8	4.2 ± 0.37	2.0 ± 0.0	3.2 ± 0.0	No inhibition
*Curtobacterium herbarum*	No inhibition	No inhibition	No inhibition	No inhibition	No inhibition
*Acinetobacter beijerinckii*	7.6 ± 0.24	12.6 ± 0.68	6.6 ± 0.81	9.6 ± 0.24	No inhibition
*Acinetobacter calcoaceticus*	18.4 ± 0.68	10.6 ± 0.51	10.6 ± 0.4	No inhibition	4.2 ± 0.37
*Achromobacter xylosoxidans*	6.8 ± 0.66	2.0 ± 0.0	No inhibition	1.0 ± 0.0	No inhibition
*Staphylococcus succinus*	10.6 ± 0.68	4.0 ± 0.0	2.4 ± 0.24	1.0 ± 0.0	No inhibition
*Staphylococcus sciuri*	2.0 ± 0.0	3.0 ± 0.0	1.0 ± 0.0	6.6 ± 0.24	1.0 ± 0.0

For evaluation of antibacterial effect of isolated strains of pure and combined essential oils, a multivariate statistical method, principal component analysis (PCA) was used. The PCA results showed that the first principal component accounted for 54.44%, the second component 30.50% and 12.59% the third component of the total variance. The first three principal components (PC) together accounted for 97.53% of the total variance.

**FIGURE 1:**
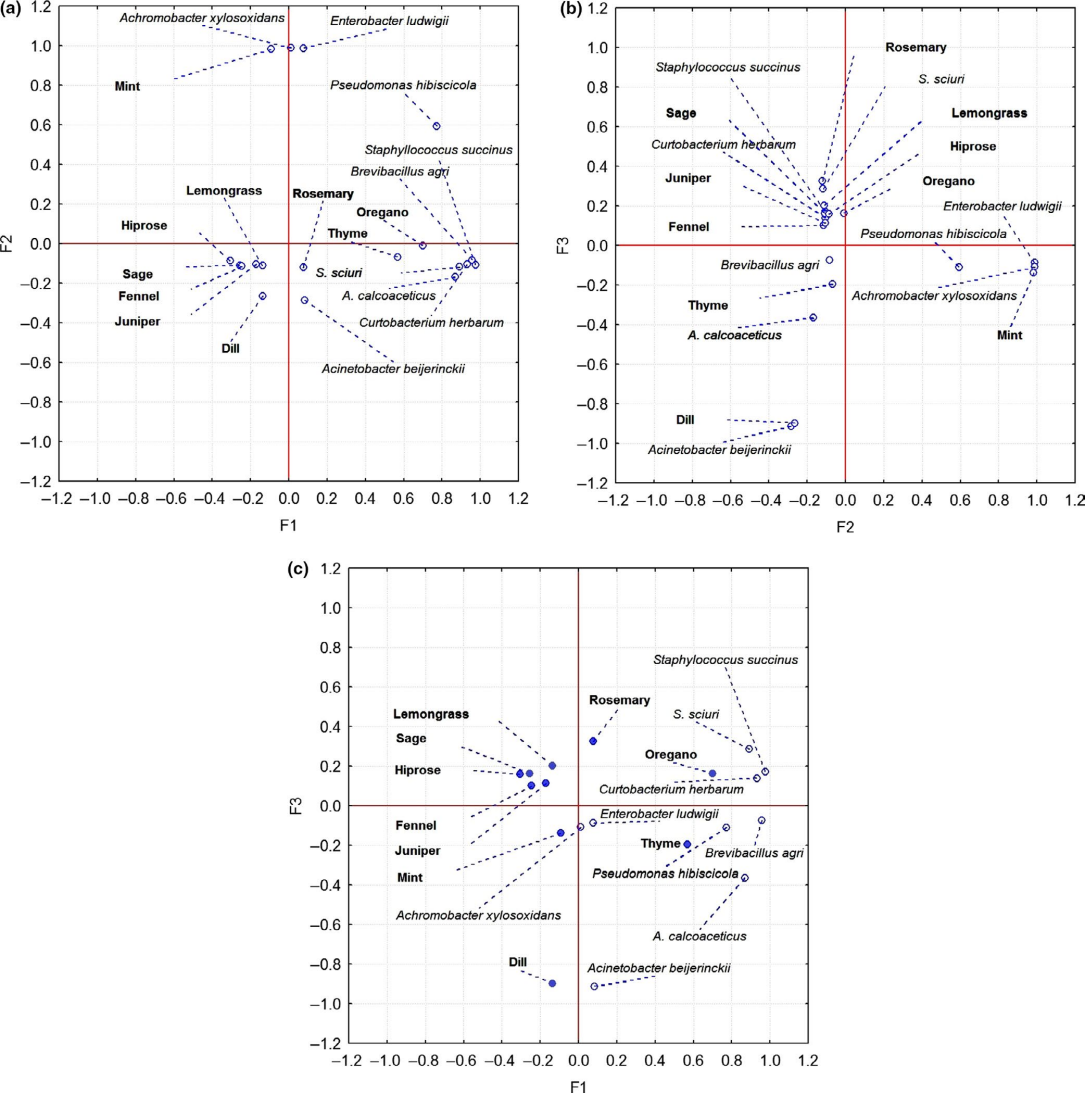
PCA biplot analysis of PCs—(a) PC1‐PC2, (b) PC1‐PC3, (c) PC2‐PC3—to elucidate the efficiency of antibacterial effects of ten essential oils on different isolated bacterial strains

According to eigenvalues, which represent the total amount of variance explained by a given principal component, the components (factor) with an eigenvalue >1.00 are retained and interpreted. The essential oil antibacterial effects on different strains are represented on the biplot analysis on the PC1–PC2, PC1–PC3, and PC2–PC3 coordinate systems (Figure 1). The PCA shows that the essential oils had a selective antibacterial effect. Oregano and thyme showed closer association and exerted the greatest antimicrobial effect on the majority of bacterial strains. It can be observed that the majority of essential oils (grouped in the left quadrant of the biplot PC1‐PC2, PCA figure (b)) exhibited low or lack of activity. The mint with three total inhibition cases is also represented on PCA figure (b). It was also shown that mint possesses good antibacterial activity against *Enterobacter aerogenes, E. cloacae*, resulting damage to membrane (Bouyahya et al., 2020; Raut & Karuppayil, 2014). The rosemary is situated in the same quadrant with oregano, possessing a high selectivity against *Staphylococcus* species and *Curtobacterium herbarum*, as well as moderate antimicrobial effect against the other isolated bacterial strains.

To evidentiate the inhibition synergy between the components of EO combinations, the inhibition zone diameter measurement results were subjected to PCA. The results showed that the first principal component (PC) accounted for 46.78%, the second 20.03%, and the third 11.43% of the total variation.

**FIGURE 2:**
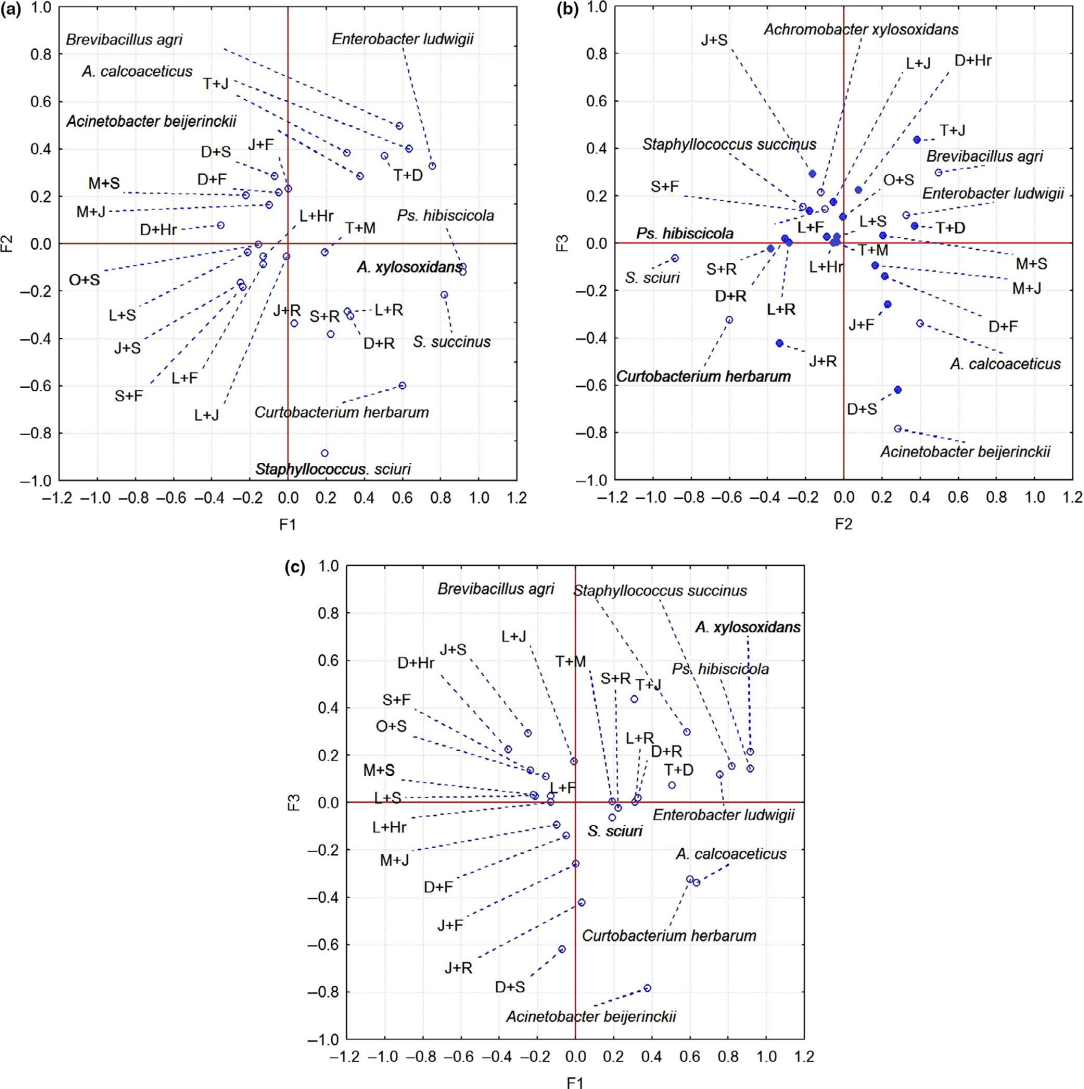
PCA biplot analysis of PCs—(a) PC1‐PC2, (b) PC1‐PC3, (c) PC2‐PC3 of the antibacterial activity of the studied essential oil combinations (L‐Lemongrass, J‐ Juniper,F‐ Fennel, S‐Sage, R‐ Rosemary, Hr‐ Rosehips, D‐Dill, T‐Thyme, M‐Mint, O‐Oregano) on isolated strains

The statistical evaluation of the antimicrobial effect of the EO combinations (expressed by inhibition zone mean diameter) resulted in the greatest sensitivity effect for T + J (thyme + juniper) and T + D (thyme + dill) combination. Based on Figure 2 (PC1‐PC2 scatterplot), other adjacent associations that present synergism are J + R (juniper + rosemary), L + R (lemongrass + rosemary), S + R (sage + rosemary), and D + R (dill + rosemary). These combinations possess synergistic antibacterial effect. The synergistic effect of rosemary, thyme with other spice essential oils was reported by other researcher too. It was observed that less‐active compounds, as in the case of rosemary, are enhanced by compounds from other EOs. The complementary chemical constituents contribute to the damage of the outer membrane or metabolic activities (García‐Díez et al., 2017; Ambrosio et al., 2019; Nikkhah, Hashemi, Habibi Najafi, & Farhoosh, 2017).

In the PC1‐PC3 scatterplot in the two right quadrants are placed the combination of EOs with only weak antibacterial effect. PC1 contains the strains *Pseudomonas hibiscicola, Acinetobacter calcoaceticus, Achromobacter xylosoxidans, Staphylococcus succinus,* and PC3 containing *Brevibacillus agri*. It was observed that the aforementioned strains were the most sensitive to EOs combinations. According to recent researches (Raut & Karuppayil, 2014; Reddy, 2019), various essential oils exhibit antibacterial activity against human pathogenic as well as food spoilage bacteria and against the isolated strains from fresh vegetable surface.

Using essential oil as antimicrobial agent (e.g., in food preservation and packaging) may decrease the risk of foodborne infections and could decrease the overuse of antibiotics, especially the emergence of antimicrobial resistance (AR). Essential oil compounds affect the bacterial cells by different mechanisms in comparison with traditional antibiotics. A synergic effect between these two compound classes may even occur. Another important strategy in the antibacterial fight is the reversing the antibiotic resistance (Kristiansen, Thomsen, Martins, Viveiros, & Amaral, 2010). The general principle of this strategy is given by the concept of collateral sensitivity, namely that microbial populations adapted to one class of antibiotics will have low fitness for one other class of antimicrobial compounds (Pál, Papp, & Lázár, 2015). A main AR mechanism is the increased activity of efflux pumps. The decreased activity of these pumps make bacteria vulnerable (Lázár et al., 2013), and active blocking of the efflux pump function or cell wall disruption is an effective way to counteract the AR (Langeveld, Veldhuizen, & Burt, 2014; Mouwakeh, Telbisz, Spengler, Mohácsi‐Farkas, & Kiskó, 2018). As these two mechanisms are very common for essential oil compounds, this could be the key for AR reversing activity of the EOs (Yap, Yiap, Ping, & Lim, 2014). The efficiency of this strategy was confirmed experimentally for Gram‐negative multidrug‐resistant strains (Lorenzi et al., 2009; Yap, Lim, Hu, & Yiap, 2013). In case of multidrug resistance, blocking the efflux pumps with essential oil compounds may prevent the ejecting of multiple drugs from microbial cells, which have lost their efficiency due by AR. Previously, the strategy of regaining antibiotic efficiency by incorporation of nonantibiotic compounds (e.g., clavulanic acid, a β‑lactamase inhibitor) was effective in the fight with AR in the case of penicillin‐resistant strains (Cheesman, Ilanko, Blonk, & Cock, 2017). Until now, only several EOs possess a demonstrated strong individual antimicrobial effectiveness and more research is needed. Even though the antimicrobial effect of the majority of EO compounds is significantly weaker compared to antibiotics, their low toxicity level, as well as their natural origin, makes them attractive in both the food and cosmetic industries (Wińska et al., 2019).

## 4 CONCLUSION

The present study has shown that different pathogenic and food spoilage bacteria occur on the surface of fresh vegetables. The antimicrobial activity of studied essential oils of locally grown spices on the growth of identified bacteria shows differences in function of bacterial strains. The highest efficiency was observed for oregano essential oil. Some of the essential oil used in our experiments contains thymol and carvacrol (oregano and thyme) as main active component, with demonstrated efflux pump inhibitor effects, that are good candidate in prevention or even reversal of antibiotic resistance. Based on the results, it can be concluded that some individual essential oils and mixture compositions have the potential for practical antimicrobial application. These could be good candidates for the preservation of fresh vegetables, decreasing the health risk of foodborne infections, and also could contribute to the suppression of antibiotic resistance.

## CONFLICT OF INTEREST

The authors declare that they do not have any conflict of interest.
